# Outstanding issues in the study of antipredator defenses

**DOI:** 10.1002/ece3.10803

**Published:** 2023-12-11

**Authors:** Yuqian Huang, Tim Caro

**Affiliations:** ^1^ School of Biological Sciences University of Bristol Bristol UK; ^2^ Center for Population Biology University of California Davis California USA

**Keywords:** antipredator, defense behavior, protective coloration

## Abstract

Protective defense mechanisms are well documented across the animal kingdom, but there are still examples of antipredator defenses that do not fit easily into the current conceptualization. They either fall within the intersection of multiple mechanisms or fail to fall neatly into pre‐existing categories. Here, using Endler's predatory sequence as a framework, we identify problematic examples of antipredator defenses, separating them into protective mechanisms that are difficult to classify and those which act sequentially depending on context. We then discuss three ways of improving underlying terminological and definitional problems: (1) issues with English and polysemy, (2) overlapping aspects of similar mechanisms, and (3) unclear definitions. By scrutinizing the literature, we disentangle several opaque areas in the study of protective defense mechanisms and highlight questions that require further research. An unclear conceptual framework for protective defense mechanisms can lead to misconceptions in understanding the costs and benefits of defenses displayed by animals, while interchangeable terminologies and ambiguous definitions can hinder communication in antipredator studies.

## Graphical abstract

Although the main forms of antipredator defenses in animals are well defined, there are still many examples of overlapping mechanisms and others that do not fall neatly into pre‐existing categories. In our review, we systematically discuss these problems and suggest ways of improving underlying terminological and definitional problems. By scrutinizing hitherto unexplored examples of confusing antipredator defenses across taxa, we disentangle opaque areas in the study of protective defense mechanisms and highlight questions that require further research.

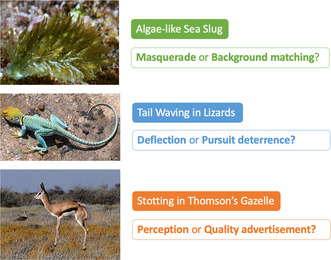

## 1 INTRODUCTION

There has been a long history of documenting protective defense mechanisms in animals (e.g., Cott, 1940; Poulton, 1890; Ruxton et al., 2018a; Wallace, 1867) which can be ordered according to where they are employed during the predatory sequence (Table 1; Endler, 1991, Ruxton et al., 2018a). At the beginning of the sequence, prey may use protective coloration such as background matching, countershading, and transparency to avoid detection by a predator. If the prey has been detected, it may try to avoid identification using disruptive or distractive markings, masquerade, or forms of mimicry. If the prey has been recognized, various protective defense mechanisms can hinder or stop the attack. Aposematism and pursuit deterrence remind the predator of the likely unprofitability of attack; deimatic displays trigger an unlearned avoidance response in the predator; and the withdrawal of the flash behavior at the end of the chase can hinder the successful location of prey. Finally, there are still protective defense mechanisms that can minimize the damage during the subjugation phase. Prey might have a deflective marking that directs the point of attack to a less vital part of the body, or might employ tonic immobility as a last resort, causing the predator to lose interest (Ruxton et al., 2018a), or possess spines, a tough exoskeleton or be toxic to impede swallowing (Caro & Ruxton, 2019).

**TABLE 1:** Prominent examples of definitions of protective defense mechanisms in the order of the predatory sequence.

Protective mechanisms	Definition(s)
Avoiding detection	
Background matching	“Where the appearance generally matches the colour, lightness and pattern of one (specialist) or several (compromise) background types” (Stevens & Merilaita, 2008, p. 424)
Countershading	“Animals are painted by nature, darkest on those parts which tend to be most lighted by the sky's light, and vice versa” (Thayer, 1896, p. 125)
Disruptive marking/coloration	“Being a set of markings that creates the appearance of false edges and boundaries, and hinders the detection or recognition of an object's, or part of an object's, true outline and shape” (Stevens & Merilaita, 2008, p. 424)
Avoiding identification	
Decoy	“A decoy object that resembles the spider and distracts a predator's attention from the nearby spider” (Eberhard, 2020, p. 169)
Distractive marking	“Direct the ‘attention’ or gaze of the receiver from traits that would give away the animal (such as the outline)” (Stevens & Merilaita, 2008, p. 424) “Distractive markings are markings that through (and despite) their relative salience compared to the rest of the coloration or morphology of an animal make it more difficult for a viewer to perceive the characteristics useful for detection or recognition of the animal, hence increasing its net camouflage” (Merilaita et al., 2013, p. e1271) “Distractive markings direct the “attention” or gaze of the receiver from traits that would give away the animal (such as the outline), or interfere with visual mechanisms that an observer could use to detect or recognize an object by virtue of the distractive markings' high‐contrast or conspicuousness” (Stevens, Troscianko, et al., 2013, p. e1273)
Masquerade	“Where recognition is prevented by resembling an uninteresting object, such as a leaf or a stick” (Stevens & Merilaita, 2008, p. 424) “One whose appearance causes its predators or prey to misclassify it as a specific object found in the environment, causing the observer to change its behaviour in a way that enhances the survival of the masquerader. Any change in the population/evolutionary dynamics of the model caused by the presence of the masquerader will not be as a result of the signal receiver changing its behaviour towards the model” (Skelhorn, Rowland, & Ruxton, 2010, p. 4) “An uncanny resemblance to inedible and often inanimate objects that are commonly found in their local environment” (Skelhorn, 2015, p. 64)
Aggressive mimicry	“A system can be classified as aggressive mimicry when the advertised benefits to the receiver are greater than the actual benefits” (Jamie, 2017, p. 4–5)
Batesian mimicry	“Members of a palatable species (the ‘mimic’) gain a degree of protection from predators by resembling an unpalatable or otherwise defended species (the ‘model’)” (Bates, 1862)
Cue mimicry	“When mimic and model have different receivers or when there is no receiver for the model's trait” (Jamie, 2017, p. 1)
Evasive/escape mimicry	“The similarity in colour, form, or behaviour that arises among species because they are difficult to capture and thereby unprofitable for the predator to pursue” (Srygley & Norton, 1999, p. 692)
Locomotor mimicry	“The similarity in swimming, walking, or flying of distantly related animals, and the associated similarity in form and physiological function” (Srygley & Norton, 1999, p. 69)
Müllerian mimicry	“Two or more species with effective defences share a similar appearance or signalling, and by this sharing reduce the cost of associative learning, and even promote the evolution of refraining from attack by their enemies” (Lev‐Yadun, 2018, p. e1480846)
Quasi‐Batesian mimicry	“If there are some levels of discrepancy in protection between two mimetic species, that the less well defended species could act in a Batesian manner, diluting the protection of a better defended species” (Speed, 1999, p. 757)
	“An undefended mimic helps rather than harms the model” (Balogh et al., 2008, p. 159)
	“A less defended mimic is more beneficial to the model than an equally defended one” (Balogh et al., 2008, p. 158)
Stopping or hindering the attack	
Aposematism	“Aposematism is the pairing of two kinds of defensive phenotype: an often repellent secondary defence that typically renders prey unprofitable to predators if they attack them and some evolved warning signal that indicates the presence of that defence” (Ruxton et al., 2018a, p. 84)
Vigilance advertisement	“Signals used by prey that advertise its vigilance towards a potential predator even if the predator has not yet been detected” (Huang & Caro, this paper)
Perceptual advertisement	“Is directed at a stalking or ambushing predator that relies on being able to gain close proximity to the prey prior to detection; the prey signals that it has been detected prior to it coming sufficiently close to mount a successful attack” (Ruxton et al., 2018b, p. 128)
Pursuit deterrence	“Signals used by prey that apparently convince predators not to pursue them” (Caro, 1995, p. 500)
Quality advertisement	“The prey signals to a coursing predator that is a particularly fleet individual, that the predator will struggle to close on, and/or a particularly strong individual, that will be difficult to subdue if the predator does succeed in closing on it” (Ruxton et al., 2018a, p. 128)
Deimatic display	“A behaviour performed by a target different from fleeing and retaliation that is triggered by it perceiving threat from an attacker during approach or subjugation, and which can trigger an unlearned avoidance response in the attacker causing it to slow or stop its attack” (Drinkwater et al., 2022, p. 4)
Flash behavior/coloration	“Sudden disappearance of colour combined with the equally sudden suspension of movement which tends to mislead the eye and to render the animal's exact whereabouts on alighting all the more difficult to detect” (Cott, 1940, p. 376) The predator “may be caused to hesitate by the sudden movement and appearance of the bright colour … and it may follow this colour and be deceived by its sudden disappearance into assuming the prey has to vanish whereas in reality the prey has come to rest in its normal cryptic posture with the coloured structures hidden” (Edmunds, 1974, p. 146) “In which otherwise cryptic prey exhibit conspicuous coloration or noise when fleeing from potential predators, has been postulated to hinder location of prey once they become stationary” (Loeffler‐Henry et al., 2018, p. 528) “By contrast, we define flash displays as a behaviour that is performed only in motion to confuse or misdirect a predator in the pursuit of prey” (Kim et al., 2020, p. 1)
Minimizing the damage	
Deflective/divertive marking	“Deflection marks direct an attack either at a non‐essential part of the body or a positively distasteful part of the body” (Edmunds, 1974, p. 176) “Markings that increase prey survival by diverting attacks towards less vital body parts or towards a direction that would facilitate escape” (Kjernsmo & Merilaita, 2013, p. 1) “Deflection involves traits that influence the initial point of contact of the predator on the prey's body in a way that benefits the prey” (Ruxton et al., 2018a, p. 189)
Tonic immobility	“Tonic Immobility (TI) is the unlearned adoption of a motionless posture by a prey individual triggered by physical contact or very close proximity of – not injury inflicted by – a predator (or other antagonist). The posture does not reduce the sensory ability of the predator to locate or identify the prey, or reduce the physical vulnerability of the prey if the attack is pursued. The state of motor inhibition is maintained for a time even after release by the predator, and when in this state the prey exhibits reduced responsiveness to external stimulation (although monitoring of the environment can still occur). In the absence of mortality or injury during TI, the prey can recover its original physiological state on emerging from TI” (Humphreys & Ruxton, 2018, p. 21)

Despite biologists having identified this sequence of protective defense mechanisms, some examples remain problematic. First, there are examples of species using defense mechanisms that are still poorly understood. They either lie in the overlap between different mechanisms or do not fit neatly into established categories, making the empirical differentiation between mechanisms difficult. Some of these difficulties arise from a lack of data; others arise from conflicting information from different sorts of experimental designs. Second, there are examples where a single morphological appearance can be effective through multiple different mechanisms depending on context. A holistic approach is needed to reveal all the possible mechanisms that a single defense could achieve.

Furthermore, the underlying etymology of some of these mechanisms also needs clarification. Specifically, these include improving poor use of English due to ambiguous terms, clearing up confusion arising from overlapping aspects of similar mechanisms, and avoiding unclear definitions. Since lack of clarity can lead to misunderstandings about predator–prey dynamics, this review attempts to expose and clarify opaque areas of our understanding of protective defense mechanisms in animals and suggests future avenues of research into animal defenses.

## 2 PROBLEMATIC ANTIPREDATOR DEFENSES

### 2.1 Protective mechanisms that lack consensus

Some protective coloration mechanisms are still poorly understood owing to lack of data or conflicting experimental results. Examples include defense behavior in the Sphinx moth caterpillars (Skelhorn, Rowland, & Ruxton, 2010), lizard tail‐wiggling behavior (Cooper, 2001; Telemeco et al., 2011; York & Baird, 2016) and deer tail‐flagging behavior (Caro et al., 1995; Stankowich, 2008) which we use to highlight the need for additional evidence to interpret underlying evolutionary mechanisms.

#### 2.1.1 Warning coloration or deimatic behavior?

**FIGURE 1:**
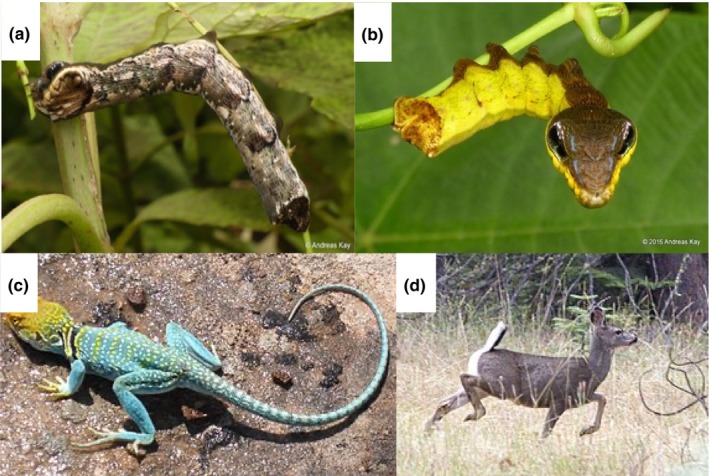
Problematic examples of species using protective mechanisms which are difficult to classify. (a) Dorsal side of *Hemeroplanes ornatus* resembling a twig for background matching or masquerade, image: Andreas Kay; (b) ventral side of *Hemeroplanes triptolemus* mimicking a snake and flattening its head as a form of deimatic behavior or Batesian mimicry, image: Andreas Kay; (c) collared lizards (*Crotaphytus collaris*) which perform tail‐waving behavior for pursuit deterrence or deflection, image: Ejohnsonboulder; (d) black‐tailed deer (*Odocoileus hemionus*) flagging its tail, signaling either to conspecifics or predators, image: Oregon Department of Fish & Wildlife.

Sphinx moth caterpillars (*Hemeroplanes triptolemus* and *H. ornatus*), also known as the “snake resembling caterpillars”, have been iconic in popularizing the field of camouflage, but surprisingly, they have yet to be studied experimentally (Figure 1a,b). On the dorsal side of the caterpillar, the patchy brown color resembles a twig or a stem. If disturbed the caterpillar will hang from the branch, flatten its head, and appear to human observers, to resemble a snake on its ventral side. This could be considered Batesian mimicry, as described by Henry Walter Bates in his encounter with a snake‐resembling caterpillar: “The most extraordinary instance of imitation that I ever met… [the caterpillar] startled me by its resemblance to a small snake.” (Bates, 1862, p. 509). Mimicking the predator's own predator may cause false identification of the caterpillar, causing a bird to break its attack and retreat. Alternatively, one might argue that this defensive posture is a form of deimatic behavior (Drinkwater et al., 2022). When the caterpillar suddenly hangs from the branch exposing an eyespot on the ventral side and widening its head, it might cause a startle response even before recognition has occurred (Hossie & Sherratt, 2013). This is similar to deimatic behavior exhibited by suddenly revealing eyespots in Lepidoptera (Drinkwater et al., 2022). Strangely, the incorporation of deimatism may impede the development of avoidance learning, and thus, may reduce the efficacy of Batesian mimicry so the mechanisms might have different consequences. Experiments on a predator's response to this defense behavior are needed (Umbers et al., 2019).

#### 2.1.2 Pursuit deterrence or deflection?

Many lizard species move their tails when they perceive a predatory threat (Figure 1c). Some species curl their tails laterally (e.g., *Leiocephalus carinatus* and *L. barahonesis*), some raise and wag tails rapidly (e.g., *Bassianna duperreyi*), and others include lateral and sinusoidal waves and tail curls (e.g., *Crotaphytus collaris*) (Cooper, 2001; Telemeco et al., 2011; York & Baird, 2016). Whether lizards' tail movements are a pursuit‐deterrent signal or a means of deflection (see Table 1), or both, has been debated for over two decades.

Difficulties in interpretation are driven in part by experimental protocol. In 10 studies (see Telemeco et al., 2011), the three that supported pursuit deterrence were derived from simulated ‘predators’ (humans) slowly but directly approaching lizards in the field. Tail‐wiggling behavior was displayed when the ‘human’ predator was at an intermediate distance, suggesting perhaps a signal of the lizard's ability to escape in time. In contrast, most studies supporting the deflection hypothesis came from exposing lizards to natural snake predators in experimental arenas in which snake attacks were oriented toward the waving tails (but see Cooper (2001) which supported both). In Cooper's study (2001), the lizards displayed tail‐curling behavior during spontaneous movement when no predator was approaching and were more likely to uncurl their tails under bushes, perhaps supporting the deflection hypothesis. But spontaneous tail‐curling may also signal alertness toward an undetected ambushing predator via vigilance advertisement (Table 1), requiring further experiments to compare the alertness of tail‐curled and uncurled lizards.

The study by Telemeco et al. (2011) was the only one that supported the deflection hypothesis employing neither a natural predator nor a human in the experimental design. Instead, the authors used a paintbrush to stimulate the tail‐waving behavior of hatchling Australian three‐lined skinks (*B. duperreyi*) and found that slower individuals waved their tails more frequently. They concluded that the behavior functioned to deflect predatory attacks to the torso. They further investigated the phylogenetic relationships among four lizard families (Iguanidae, Lacertidae, Scincidae, and Gekkonidae) and found that the deflection hypothesis is supported in all four of them, but only two families, Iguanidae and Gekkonidae, provided evidence supporting the pursuit deterrence hypothesis. Thus, it is likely that the deflection hypothesis is the ancestral function, and that pursuit deterrence is the less common derived function.

More recent studies of tail‐waving behavior in collared lizards (*C. collaris*; Figure 1c) and curly‐tail lizards (*Leiocephalus* spp.) cannot distinguish between pursuit deterrence or deflection when approached by humans because their findings support both hypotheses (Kircher & Johnson, 2017; York & Baird, 2016). Investigating the rate and position of attack of potential predators (e.g., birds or snakes) toward a mechanical model lizard of different sizes with different levels of the tail‐waving display could provide useful insights into this debate.

#### 2.1.3 Quality advertisement or alarm signaling?

The function of tail‐flagging behavior in deer is enigmatic (Caro et al., 1995). Some work suggests that tail‐flagging signals quality advertisement (see Table 1) of the prey's ability to escape danger, as flagging deer flee at greater speeds than non‐flaggers (Caro et al., 1995; Figure 1d). A phylogenetic comparative study, however, found that tail‐flagging behavior in ungulates is strongly associated with being attacked by stalking predators and may therefore inform ambushing predators that they have been seen (see Table 1) (Caro et al., 2004). Disconcertingly, this second study also found artiodactyls living in intermediate‐sized groups or which inhabit open environments are more likely to flag their tails. This supports the hypothesis that tail‐flagging behavior is a form of intraspecific communication, also reinforced by an observational study suggesting that tail‐flagging may convey potential danger to conspecifics (Stankowich, 2008). The difference in the quality advertisement versus the alarm signaling purpose of tail‐flagging affects our interpretation of whether this is selfish behavior or evolves through kin selection.

An added problem is the use of different terms when describing tail movements. For example, tail‐flagging has been used interchangeably with tail‐flicking, tail‐swaying and tail‐wagging in literature (Camerlink & Ursinus, 2020; Stankowich, 2008) but several functions may be involved depending on the type of tail movement. Consistent use of descriptive terminology is an important first step to establishing a clearer understanding of the protective mechanisms.

Overall, these problematic examples demonstrate that lack of experimental evidence can lead to conflicting interpretations. Further understanding of these protective mechanisms and their ecological implications necessitate rigorous experimental studies, precise terminological frameworks, and detailed examination of predator responses.

### 2.2 Species using multiple defense mechanisms depending on the context

Depending on context, some species are able to use the same appearance for different defense mechanisms. For instance, depending on the background, some can switch between masquerade and background matching (Bu et al., 2020); conditional on viewing distance, some may switch between crypsis and aposematism (Barnett & Cuthill, 2014); and depending on the predator's toxin tolerance, some may switch between Batesian and Müllerian mimicry (Rowland et al., 2010; Speed et al., 2000). Here, we discuss three instances of context‐dependent defense mechanisms and in so doing highlight the importance of using modern technology to provide a more holistic approach when investigating protective coloration mechanisms.

#### 2.2.1 Background context

**FIGURE 2:**
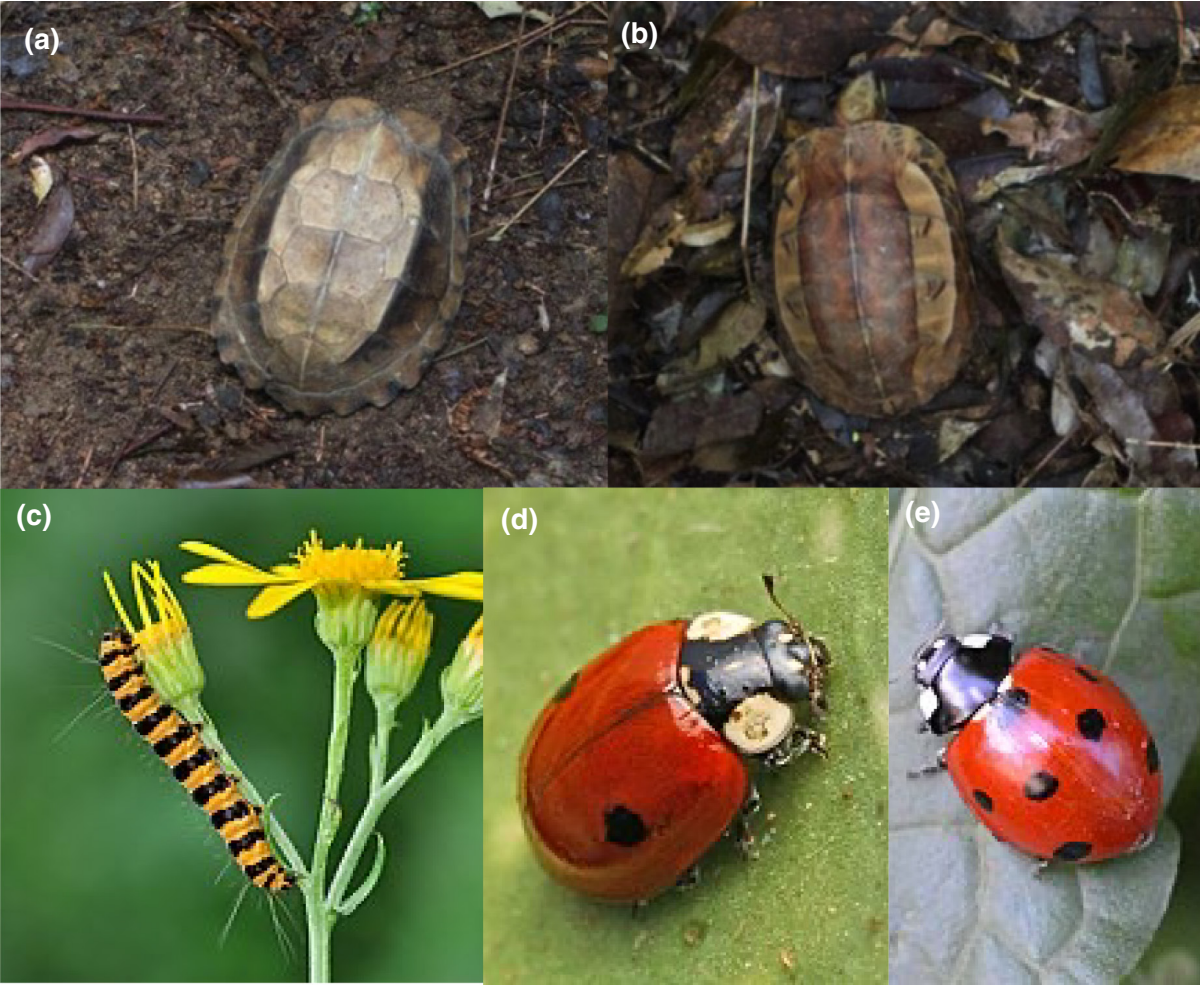
Problematic examples using of species using multiple defense mechanisms act in turn depending on the context. Carapace of (a) Indochinese box turtle (*Cuora galbinifrons*) and (b) keeled box turtle (*C. mouhotii*) resembling leaves found on their preferred leaf litter, images: with permission from Bu et al. (2020); (c) cinnabar moth caterpillar (*Tyria jacobaeae*) achieving both distance‐dependent camouflage and aposematism, image: Quartl; (d) red form of the two‐spot ladybird (*Adalia bipunctata*) resembling its more toxic model, (e) the seven‐spot ladybird (*Coccinella septempunctata*), images: Entomart and Dimitar Boevski.

To maximize the effectiveness of camouflage when moving around, some animals employ different protective coloration mechanisms according to their background. The Indochinese box turtle (*Cuora galbinifrons*) and keeled box turtle (*C. mouhotii*) have yellow stripe(s) on their dark brown carapaces, for example (Figure 2a,b, respectively). These color patterns could serve several protective mechanisms, including disruptive coloration, background matching, and masquerade of common leaves found in their preferred leafy substrate (Bu et al., 2020, 2022). If the entire carapace is exposed in an open habitat, then the brighter two yellow stripes on *C. galbinifrons* and one large yellow patch on *C. mouhotii* appear to masquerade as leaves. If the turtles half‐bury themselves in leaf litter with only the middle stripe(s) exposed, then these stripes will likely match the leaf litter background (Bu et al., 2022). The turtle's outline may additionally be disrupted by the strong contrast between the color patches on the carapace. Thus, species could be using multiple defense mechanisms together thereby maximizing survival in a heterogeneous environment. This makes it difficult to categorize their appearances easily.

#### 2.2.2 Distance‐dependence

Protective coloration can be distance‐dependent, achieving both camouflage and aposematism to increase the benefit beyond that of using only one mechanism (Barnett & Cuthill, 2014). The cinnabar moth caterpillar (*Tyria jacobaeae*) is a brightly colored toxic caterpillar that has alternating orange‐and‐black horizontal stripes on its body (Barnett, Cuthill, & Scott‐Samuel, 2018; Figure 2c). At greater distances, these orange‐and‐black stripes merge (when viewed under an avian visual model), providing an overall brown coloration and reducing the distinction between the caterpillar and its background. But at a close distance, the caterpillar is easily detectable from the background across all luminance and color channels (Barnett, Michalis, et al., 2018; Barnett, Cuthill, & Scott‐Samuel, 2018). Therefore, these caterpillars could benefit from background matching to avoid initial detection by avian predators and reduce the rate of attack. Once detected, the conspicuous orange‐and‐black signals will act as an aposematic signal, reminding the predator about its unpalatability. Similar distance‐dependent camouflage and aposematism have been shown in swallowtail butterfly larvae (*Papilio machaon*; Tullberg et al., 2005), European vipers (*Vipera* spp.; Valkonen et al., 2011), poison dart frogs (*Dendrobates tinctorius*) using model prey (Barnett, Cuthill, & Scott‐Samuel, 2018) and the spotted skunks (*Spilogale gracilis*; Caro et al., 2013). Thus, the same morphological appearance achieves two distinct protective coloration mechanisms making it difficult to categorize defense mechanisms easily. Future research might investigate other environmental conditions aside from viewing distance that can influence the exact protective mechanism in use, such as lighting conditions and habitat contexts.

#### 2.2.3 Toxin‐tolerance dependence

Protective coloration mechanisms can also change contingent upon the predator's tolerance of a toxin. Predators with a higher tolerance may be able to gain nutrients from consuming the prey and consider it profitable compared to predators with a lower toxin tolerance. This generates debate about protective coloration mechanisms when a moderately unpalatable prey's coloration mimics a highly unpalatable species – should this be considered as Müllerian, Batesian, or quasi‐Batesian mimicry. Quasi‐Batesian mimicry (Speed, 1993, 1999) refers to a less unpalatable Müllerian mimic acting in a Batesian manner and diluting the protection gained from the appearance of a better‐defended species. The presence of a moderately unpalatable mimic may incur greater per capita mortality of the more unpalatable model and result in a more parasitic relationship (Rowland et al., 2010; Speed et al., 2000).

In short, quasi‐Batesian mimicry can be considered on a continuum between Müllerian and Batesian mimicry, with the exact position dependent on the predator. For example, the two‐spot ladybird (*Adalia bipunctata*) exists in two forms, with the red form mimicking the seven‐spot *Coccinella septempunctata* and the melanic form mimicking the four‐spot *Exochomus* sp. (Brakefield, 1985; Figure 2d,e). Feeding experiments with nestling blue tits (*Cyanistes caeruleus*) have shown that the red two‐spot *A. bipunctata* (mimic) is only mildly toxic compared to the highly toxic seven‐spot *C. septempunctata* (model), confirming the quasi‐Batesian nature of the mimicry (Marples et al., 1989). However, nestling blue tits that fed on the red two‐spot mimic showed no difference in weight gain compared to a non‐toxic control group, whereas the seven‐spot model killed the nestlings within 2 days (Marples et al., 1989). This suggested that the two‐spot mimic acted mostly like a Batesian mimic of the seven‐spot model for the nestlings. However, nestlings that fed on the red two‐spot mimic begged for more additional food. This could indicate slight unpalatability of the mimic compared to the model despite no difference in profitability in terms of nutrients gained. Furthermore, predators of the ladybirds may differ in their toxicity tolerance, and thus, for a predator with lower toxin tolerance, the two‐spot mimic could be categorized as a Müllerian mimic. Predators' toxin tolerance and a mimic's toxicity may also change due to temporal and environmental factors, such as the age of the predator, predator hunger, and availability of the food that provides the toxin (Kaczmarek et al., 2020). Future studies may need to incorporate a more all‐inclusive approach with the combination of chemical analysis, behavioral assessments of the responses of the most significant predator, and the costs of the putative models for each system in order to better assess the degree and type of mimicry involved.

To summarize, understanding the context‐dependent nature of protective coloration is important. Species can switch between different defense mechanisms based on circumstances highlighting the dynamic nature of evolution and the coevolutionary arms race between predators and prey. To further complicate the story, prey may also evolve multiple defenses. These may be favored if each defensive mechanism is targeted toward a different enemy or used at a different stage of the predator–prey interaction, and these may also be constrained by resource trade‐offs (Kikuchi et al., 2023). Comprehensive studies of species' defenses operating through different mechanisms depending on background, viewing distance, predator's toxin tolerance level, predator involved, and trade‐offs between mechanisms will help elucidate the pressures shaping the evolution of antipredator defenses in animals.

## 3 IMPROVING CONCEPTUAL UNDERSTANDING

Although understanding of antipredator mechanisms can often be resolved with further experiments, some require improved terminology. Some of these are superficial and can be easily fixed, such as poor use of English leading to misconceptions about protective coloration mechanisms. Other misconceptions can arise from similar mechanisms being incorrectly identified. In yet other cases, definitions are still being developed and will need further discussion and resolution.

### 3.1 Issues with English

Difficulties arise from loose English due to polysemy, where a word has multiple possible meanings. This is often because the word used to describe a protective coloration mechanism has alternative meanings that are used in another context (e.g., flash and flash behavior) leading to misclassification. This section identifies three exemplar polysemies and suggests alternative words to use.

#### 3.1.1 Flash behavior

Colloquially, as well as more generally in behavioral biology, flash behavior could mean the sudden or intermittent exhibition of coloration. Indeed, flash as a verb simply describes an intermittent display of coloration by a prey (Goulet et al., 2020; Mäthger et al., 2012; Murali, 2018). More specifically in the study of protective defense in animals, however, it is defined as: “in which otherwise cryptic prey exhibit conspicuous coloration or noise when fleeing from potential predators, has been postulated to hinder location of prey once they become stationary” (Loeffler‐Henry et al., 2018, p. 528; see Table 1). The specific definition of flash behavior emphasizes coloration being displayed when prey is in motion but being terminated at the end of the flight and thereby hindering the follow‐up search. Thus, for (specific) flash behavior to operate, a prey does not need to be intermittently flashing during motion. Indeed, an intermittently flashing prey experiences no difference in survival chances compared to a continuously flashing prey in a human‐participated computer game (Loeffler‐Henry et al., 2018). In short, the use of the word “flash” in flash behavior may incorrectly imply that all displays in this protective mechanism involve intermittent exposure of color, which is not necessarily the case. We suggest, to avoid confusion, future researchers are more explicit when defining flash behavior by describing exhibition of a conspicuous color patch more precisely during motion.

**FIGURE 3:**
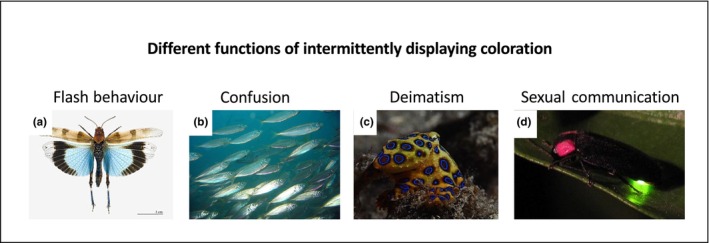
Different functions of intermittently displayed coloration used by animals. (a) Flash behavior in *Oedipoda caerulescens* when it shows its blue underwings in a deimatic display or as flash behavior, image: Didier Descouens; (b) confusion effects in schools of jack mackerel (*Trachurus declivis*), image: Richard Ling; (c) deimatic behavior, using intermittent blue coloration in Blue‐ringed octopus (*Hapalochalena lunulata*), image: Rickard Zerpe; (d) sexual communication using bio‐illuminance in fireflies (*Luciola lateralis* and *Luciola cruciata*), image: yellow_bird_woodstock.

Additionally, the term “flash behaviour” can be mistaken with other behaviors which evolved under different selection pressures (Figure 3). For example, the flashing bio‐illuminance produced by fireflies at night is used for sexual communication: the female Asian firefly (*Luciola parvula*) uses a flashing coloration at the rear of her abdomen to attract males rather than a way of responding to predators (Figure 3d; Takatsu et al., 2012). As pointed out by Loeffler‐Henry et al. (2018, p. 528), flash behavior “has been used rather broadly to represent almost any conspicuous display of color”. Other defense mechanisms can also involve color flashes too. For example, the blue‐ringed octopus (*Hapalochalena lunulata*) flashes iridescent blue coloration when disturbed which has been termed a “flash mechanism” (Figure 3c; Mäthger et al., 2012), and the lantern fish (*Myctophidae*) emits bioluminescent flashes when predatory seals are present (Barnes & Case, 1974; Goulet et al., 2020). The blue‐ringed octopus may be signaling to indicate its high toxicity, and thus, the actual defense mechanism could be aposematism, with flashing promoting conspicuousness and predator learning but unlikely to subsequently hinder location. Similarly, the lantern fish may be flashing to startle the predatory seal which slows its attack, and thus, the mechanism could be deimatic behavior. In another example, flashing in schools of jack mackerel (*Trachurus declivis*) is achieved through light reflection and acts to reduce the chance of a successful attack by the predator on any one individual (Figure 3b; Murali et al., 2019). It is worth noting that acoustic ‘flashing’ can occur during movement but may not be related to flash behavior. For example, the tiger moth (Arctiidae) produces bursts of ultrasonic noises toward predatory bats in order to warn the bats of their unpalatability (Hristov & Conner, 2005). Since flashing behavior is widespread in animals, when considering the protective defense mechanism, it is important to define it in future studies as a conspicuous coloration or noise that is only displayed in motion and will “hinder location of prey once they become stationary” (Loeffler‐Henry et al., 2018, p. 528).

Finally, flash coloration should not be interchangeable with flash behavior. The original term proposed by Edmunds (1974) was flash behavior, but later literature uses flash coloration (Caro et al., 2020; Loeffler‐Henry et al., 2018; Murali, 2018). There are two reasons why using these two terms interchangeably can lead to difficulties. First, as discussed above, flash coloration, or intermittently displayed coloration, could be involved in many other functions other than the specific form of flash behavior. Second, flash behavior may involve other conspicuous signals utilized during the escape so as to mislead the predator, such as a conspicuous noise. For example, many grasshoppers buzz or make a clicking noise when they escape from their predator alongside the display of conspicuous coloration on their hindwings (Edmunds, 1974). Thus, it is best to avoid using the term “flash coloration”.

#### 3.1.2 Distraction and deflection

Distractive and deflective markings (see Table 1) are two antipredator defenses that lack clear definitions, and consequently, there are examples that could fit into either category, and this causes them to sometimes be used interchangeably.

The definition of distractive markings has been debated, updated, and expanded several times in the past decade. The most general definition by Stevens and Merilaita (2008, p. 424) broadly defined distractive markings as any marking that could “direct the attention or gaze of the receiver from traits that would give away the animal”, and lacks a morphological description. A later definition by Stevens et al. (2013, p. 213) emphasized that distractive markings should “comprise colors not found in the background or have contrasts in excess of features of the background”. Therein lies a contradiction: conspicuous markings that are not found in the background may be used as a unique cue for the predator's search image (Cuthill, 2019). This may be the reason why visual search experiments with humans showed that conspicuous marks could be learnt and that they enhanced the detectability of prey (Stevens, Marshall, et al., 2013). Note that irrelevant conspicuous markings in the background could therefore interfere with learning about conspicuous prey markings preventing the predator from using those markings as a unique cue in searching for prey (e.g., Dimitrova et al., 2009).

While the definition of distractive markings has been expanded to include markings that are just relatively more “salien[t] compared to the rest of the coloration” (Merilaita et al., 2013, p. 1271), the word “salient” is a polysemy that can be viewed by people differently. For example, a vision biologist might define salience quantitatively and measure it using color metrics or pattern differences between an animal and its background. However, for a computational biologist, salience is a visual difference metric. Salience is also used in everyday speech to mean “relevant” or “important”, which are cognitive factors rather than the appearance of the stimulus. Therefore, using a polysemy to define distractive markings could lead to disparity in the assessment of the marking. We advocate Stevens and colleagues' definition that includes the effect of such markings on the predator, focuses on the visual mechanisms for how distraction could work, and also provides a brief description of the markings: “Distractive markings direct the “attention” or gaze of the receiver from traits that would give away the animal (such as the outline), or interfere with visual mechanisms that an observer could use to detect or recognize an object by virtue of the distractive markings' high‐contrast or conspicuousness” (Stevens, Troscianko, et al., 2013, p. 1273).

Deflective markings operate during the approach phase of the predation sequence and emphasize the redirection of the attack by the predator “either at a non‐essential part of the body or a positively distasteful part of the body” (Edmunds, 1974, p. 176). Nonetheless, such a deflective marking often “distracts” the predator's attention first before causing it to divert its attack point. Though Edmunds' definition of deflective marking is accurate, it is important to separate these terms, considering whether it is the search for the prey that has been impeded or the attack location that has been redirected. It is also best to avoid using “distract” when writing about deflective markings, and use other synonymous, less ambiguous verbs such as “divert” or “attract”.

#### 3.1.3 Decoys

A decoy (see Table 1) is another mechanism different from both distractive and deflective markings and should not be used interchangeably with them. Some species of the orb spider (*Cyclosa* spp.) incorporate egg sacs or detritus into silk decoration on their webs. For example, the orb spider (*Cyclosa mulmeinensis*) uses its prey pellet or egg sac to decorate its web which may increase its survival chances (Tseng & Tso, 2009). Decoys are similar in size to individual spiders, and their presence has led to a reduction in the proportion of overall attacks by wasps toward the spiders from 75% to only ~32%. The decoy hypothesis, described by Eberhard (2020, p. 169), suggests that these decorations “form a decoy object that resembles the spider and distracts a predator's attention from the nearby spider” (see Table 1). However, decoys are not part of the spider's body, so they should not be classified as a distractive marking to the predator (Wang et al., 2022). And since it diverts attack to an area that is not part of the spider's body, it should not be considered a deflective marking either. It may be more helpful to think of decoys as a special case of mimicry.

Overall, this section addresses several issues related to the usage of polysemy in the study of protective defenses. Words such as “flash” in flash behavior can have multiple meanings and lead to misclassification. and using “distractive” and “deflective” markings and decoys interchangeably should be avoided; explicit definitions are important.

### 3.2 Overlapping aspects of protective coloration mechanisms

Although many definitions of some protective coloration mechanisms are already clear and precise, misconceptions may still arise from the overlap between similar mechanisms. This section points to the similarities and differences of three protective coloration mechanisms.

#### 3.2.1 Deimatic behavior

Both deimatic behavior and aposematism can utilize bright coloration; indeed, deimatism may be an evolutionary precursor of aposematism (Loeffler‐Henry et al., 2023). For example, the long exposure of the conspicuous ventrum of several amphibian species upon disturbance has been historically thought of as aposematism (Wells, 2007) but is now being considered as deimatic behavior (Chiocchio et al., 2023). One crucial difference is that although deimatic behavior may vary greatly in the performance duration, its main goal is to “trigger an unlearned avoidance response in the attacker” (Drinkwater et al., 2022, p. 4), whereas aposematism is often, but not always, displayed to enhance the avoidance learning process in the attacker. The former requires the process of predator learning but the latter does not. Thus, although deimatic behavior can include an aposematic signal, it may restrain the development of avoidance learning and reduce the efficacy of the aposematic signal (Drinkwater et al., 2022). As articulated by Skelhorn et al. (2016, p. 2), we should “focus on the mechanisms through which they prevent predator, rather than by the form [or duration] taken by the display”, due to the large variety of deimatic displays and its potential overlap with aposematism.

Deimatic behavior may also be confused with flash behavior. First, both mechanisms involve suddenly revealing conspicuous color patches by normally cryptic animals, and have thus sometimes been used interchangeably (e.g., Kim et al., 2020). For example, the dead‐leaf mantis (*Deroplatys* sp.) reveals its conspicuous black and white underwings in its defensive posture when it perceives a threat which is considered as deimatic behavior (Drinkwater et al., 2022; O'Hanlon et al., 2018), whereas revealing the bright yellow underwing of the grasshopper (*Trilophidia tenuicornis*) during flight is considered flash behavior (Figure 3a; Cott, 1940; Edmunds, 1974). Secondly, the survival benefits of both deimatic and flash behavior may derive from predators learning to associate hidden colors with unprofitability (Kim et al., 2020). For example, more experienced domestic chicks rejected eating a mealworm which had earlier caused a startle response (unprofitability), and fewer human participants chose to follow prey with a bright color patch fleeing which has been previously associated with being “hard‐to‐chase” (Kim et al., 2020). Finally, some might argue that revealing bright coloration in both deimatic and flash behavior may cause a surprise and/or startle response (Cott, 1940; Edmunds, 1974). However, since flash behaviors tend to operate best when the predator is far away (Loeffler‐Henry et al., 2021), and the startle reflex is mostly seen when the predator is close up, it is unlikely that flash behavior would also serve a startle function. Moreover, fitness consequences differ too: compared to flash behavior, deimatism does not involve using energy to escape but could involve greater predation risk if the defense fails in proximity to the predator. As suggested by Skelhorn et al. (2016), one should focus more on the effect that the behavior has on the predator, whether it is to mislead the coursing predator about the hidden location of the prey (flash behavior) or cause the predator to misclassify the prey as a potential threat (deimatic behavior), rather than focusing on the exact form of behavior.

#### 3.2.2 Tonic immobility

Tonic immobility, also known as thanatosis or death feigning has been defined as “the unlearned adoption of a motionless posture by a prey individual triggered by physical contact or very close proximity of – not injury inflicted by – a predator (or other antagonist)” (Humphreys & Ruxton, 2018, p. 21; see Table 1). This broad definition has overlapping aspects with two other defense mechanisms – freezing and aggressive mimicry. Freezing is when the individual “stops all movements upon encountering a predator or danger and the absence of movement is maintained until the threat is gone” (Sakai, 2021, p. 10). It is a protective defense mechanism that also involves the immobility of the prey under threat. The difference between tonic immobility and freezing is that freezing occurs much earlier in the predation sequence (e.g., during the detection and recognition phase), whereas tonic immobility is used as a last resort during the attack and subjugation phase. For example, the solitary desert locust (*Schistocerca gragaria*) exhibits a catalepsy phase 10 times longer than the aposematic gregarious morphs and is thought to enhance camouflage efficacy in the solitary state. This catalepsy should, therefore, be considered as freezing, not tonic immobility since it operates during the detection phase of the predation sequence (Rogers & Simpson, 2014). Tonic immobility may also be targeted at specific predators (e.g., smaller Japanese pond frogs, *Pelophylas nigromaculatus*, find it difficult to swallow grasshoppers exhibiting tonic immobility compared to larger frogs; Sakai, 2021), whereas prey that only freezes may not even encounter the subjugation phase, and thus, be subject to different predator–prey dynamics. Not only is it important to clearly separate tonic immobility from freezing but to avoid using the word “freeze” when it only involves tonic immobility.

Some predators may also pretend to be dead to lure prey to them. Instead of tonic immobility, this should be considered as aggressive mimicry, which is “when the advertised benefits to the receiver are greater than the actual benefits” (Jamie, 2017, p. 4–5; see Table 1). Here, immobility here has evolved to enhance hunting success, rather than minimize predation. For example, two species of predatory cichlid fish (*Parachromis friedrichsthalii* and *Haplochromis livingstoni*) have been described as showing death/illness feigning and attacking small scavenging fish when they lower their guard (McKaye, 1981; Tobler, 2005). The cichlids are not prey entering an unconscious motionless posture triggered by a predator, and thus their behavior should not be categorized as tonic immobility.

#### 3.2.3 Locomotor and evasive mimicry

Two types of mimicry involving movement of the model – locomotor and evasive mimicry –can be easily confused with each other. Locomotor mimicry is a subtype and extension of classical Batesian mimicry based on similarities in movement between the model and mimic. In locomotor mimicry, the palatable mimic resembles not only the morphology of the unpalatable model, but also its escape movement (swimming, walking, or flying; Srygley, 1994; Srygley & Norton, 1999). Experimental evidence provided by Kitamura and Imafuku (2010) found that the mimetic form of palatable *Papilio polytes* (the *polytes* form) flew with a minimum positional angle and wingbeat frequency more similar to the unpalatable *Pachliopta aristolochiae* (model) than its non‐mimetic form (the *cyrus* form). Similarly, an ant‐like resemblance is seen in over 13 spider families, including mimicry in both morphology and behavior (Cushing, 1997; Subramaniam et al., 2022). Jumping spiders (*Myrmarachne formicaria*) which lack chemical defense traits mimic the walking behavior of chemically‐defended ants (*Formica* sp.) and also demonstrate their characteristically short pauses (Shamble et al., 2017). Mimicking both the appearance and movement of the model likely interferes with the receiver's species recognition.

Evasive mimicry, also known as escape mimicry, is where a mimic resembles the morphology of a model rendered unprofitable because it is difficult to catch (Srygley & Norton, 1999). Evasive mimicry could be either Müllerian, where the mimic is also hard to catch or Batesian, where the mimic is actually easy to catch. Different from locomotor mimicry, the palatabilities of the model and mimics in evasive mimicry are not considered; unprofitability arises from the predator wasting energy in pursuing them. For example, several Neotropical butterflies have bright blue or creamy bands on their wings, and this similarity in coloration has been found to signal their evasive ability to escape predators in a Müllerian mimicry ring (Pinheiro & Freitas, 2014). Naïve blue tits, *C. caeruleus*, are also able to generalize across wing patterns of evasive *Adelpha* butterflies which are difficult to capture (Páez et al., 2021). Similarly, relatively slow‐moving weevils (*Timorus sarcophagoides*) bear a highly similar color pattern as the evasive flesh fly suggesting Batesian evasive mimicry underlies the resemblance (Guerra, 2019).

Overall, both locomotor and evasive mimicry involve the movement of the mimic or model, which can easily lead to confusion between the terms. Evasive (escape) mimicry encompasses mimicking the visual appearance of the hard‐to‐catch model, whereas locomotor mimicry focuses on mimicking the typical escape behavior of an unpalatable model. Unprofitability in evasive mimicry arises from the difficulty of catching the prey, whereas the unprofitability in locomotor mimicry comes from the distastefulness of the model.

In summary, this section explores overlapping aspects of different protective coloration mechanisms while highlighting their distinctions. To reduce confusion in terminology, we emphasize the need for precise definitions and accurate categorization.

### 3.3 Unclear definitions

There are unclear definitions of protective defense mechanisms that need attention. Here, we try to identify some grey areas.

#### 3.3.1 Pursuit deterrence, vigilance advertisement, perception advertisement and quality advertisement

Pursuit deterrence has been defined as “signals used by prey that apparently convince predators not to pursue them” (Caro, 1995, p. 500; see Table 1). It can be broken down into three further defense mechanisms—vigilance advertisement, perception advertisement and quality advertisement (Caro, 2005; see Table 1). Conceivably, a single defense behavior could function through all these three defense mechanisms ordered in a predator attack sequence, starting with vigilance advertisement before a predator is detected, perception advertisement toward an (ambushing) predator prior to the attack, and quality advertisement toward a (coursing) predator during the chase.

Vigilance and perception advertisement both involve the prey signaling alertness, but a predator need not be detected during vigilance advertisement. For example, ground squirrels (*Otosphermophilus beecheyi*) display tail‐flagging behavior that is both related to their alertness and the likelihood of a snake's presence (Putman & Clark, 2015). Squirrels' tails are flagged when they are in a habitat where recent snake encounters have occurred without the snake's presence (although even more when a rattlesnake is detected). A snake is more likely to abandon its ambushing site and less likely to attempt to strike when a squirrel is advertising its vigilance (Barbour & Clark, 2012). Thus, this tail‐flagging behavior performed by squirrels is functioning both as a vigilance advertisement signal when no predator is around, and as a perceptual advertisement signal when the predator has been detected.

In comparison, perception and quality advertisement are both directed toward the predator during a later stage of the predatory sequence, and the former advertises that the stalking or ambushing predator having been detected, whereas the latter advertises the condition of the prey toward a coursing predator (Ruxton et al., 2018a). A classic example of a perception advertisement that signals the detection of an ambushing predator is the upright stance of hares (*Lepus europaeus*) shown toward foxes (*Vulpes vulpes*) (Holley, 1993). This behavior was performed on 31 out of 32 occasions when the fox was within 20–50 m of the hare, and none of these 31 ‘signaling’ hares was attacked. Yet when encountering a coursing predator such as a dog, no signaling behavior was observed. An upright stance would be considered a perception advertisement that is a directed signal and that deters ambushing predators which rely on getting close to the prey without being detected.

At the other end of the spectrum, pursuit deterrent signals acting through quality advertisement will often involve energetic costs, such that only the fitter individuals are able to display the signal. For example, Cresswell (1994) noted that non‐ or poorly singing skylarks (*Alauda arvensis*) were chased by merlins (*Falco columbarius*) for longer periods of time and were more likely to be caught compared to skylarks that sang well. Although Cresswell did not directly test the energy cost of singing in skylarks, other studies have shown that singing during flight is energetically demanding in both skylarks and other birds (Moller, 1991). Furthermore, skylarks only sang when a merlin had initiated the attack; convincing evidence that this behavior is a quality advertisement signal.

There are other more challenging examples of pursuit deterrence which are harder to classify into either perception or quality advertisement. For example, many ungulates stot toward the predator, and stotting in Thomson's gazelles (*Gazella thomsonii*) serves as a perception advertisement toward ambushing predators (Caro, 1986), yet Fitzgibbon and Fanshawe (1988) found that stotting in Thomson's gazelles is also an honest signal of condition because it occurs more in the wet season, and coursing predators such as hunting dogs (*Lycaon pictus*) are less likely to chase after gazelles that stot.

As such, we suggest that pursuit deterrence should be viewed as “a continuum of increasing costly behavior used by the prey in order to convince predators not to pursue them, with vigilance advertisement signalling to all potential predators, perception advertisement mainly targeted towards ambush predators, and quality advertisement towards coursing predators” (adapted from Caro, 1995, p. 500; Table 2). While Table 2 includes a ranking of different pursuit deterrent signals from low to high cost, it is only an estimate, as most studies rarely test the physiological cost of signaling. Thus, future studies should try to separate pursuit deterrence into perception or quality advertisement by focusing on both the energetic cost of signaling and predatory responses.

**TABLE 2:** Examples of pursuit deterrence involving increasing energetic costliness of signaling and their classification into perception or quality advertisement.

Example of pursuit deterrence	Vigilance advertisement, perception advertisement, or quality advertisement	
Tail wagging in white wagtails	Vigilance advertisement^1^	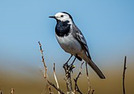
Tail‐flagging in ground squirrels	Vigilance and perception advertisement^2^	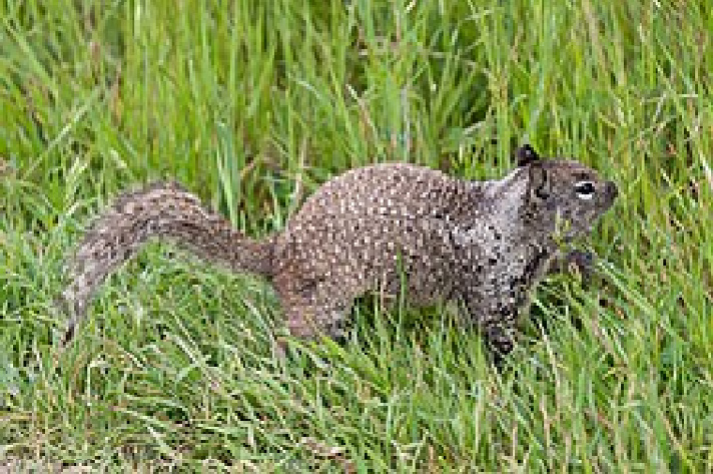
Upright stand‐by hare	Perception advertisement^3^	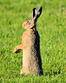
Vervet monkey call	Perception advertisement^4^	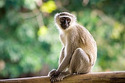
Tail‐flagging in deer	Perception and quality advertisement^5^	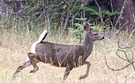
Stotting in Thomson's gazelle	Perception and quality advertisement^6^	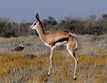
Guppy inspection	Quality advertisement^7^	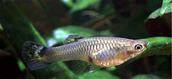
Push up, tail waving and calling behavior in lizards	Quality advertisement^8^	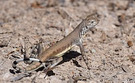
Skylark singing	Quality advertisement^9^	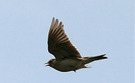

*Note*: Images by: Stephan Sprinz, King of Hearts, X posid, Thomas Shahan, Oregon Department of Fish & Wildlife, Hans Stieglitz, Holger Krisp, Andy Reago & Chrissy McClarren, and Ken Billington. References: ^1^Randler (2006), ^2^Barbour and Clark (2012), ^3^Holley (1993), ^4^Isbell and Bidner (2016), ^5^Caro et al. (1995), ^6^Caro (1986) and Fitzgibbon and Fanshawe (1988), ^7^Godin and Davis (1995), ^8^Hasson et al. (1989), Cooper (2001), Baeckens et al. (2019), and ^9^Cresswell (1994).

#### 3.3.2 Masquerade, mimicry, and background matching

Masquerade, mimicry, and background matching all involve the resemblance of another object, species, or background to reduce detection or recognition. While logically distinct (Table 1), problems arise when an animal resembles part of a larger organism. For example, the sea slug *Caliphylla mediterranea* (Costa, 1867) is a species in the *Sacoglossa* group that resembles the *Bryopsis* toxic algae (Hamann et al., 1996). This species camouflages itself from predatory fish and crabs within the algae upon which it feeds, by using flaps of its body which resemble the filaments of the algae. Krug et al. (2018) mentioned *C. mediterranea* as “(an)other striking algal mimic in Sacoglossa” and inferred it to be an example of Batesian or Müllerian mimicry. However, it is possible to consider *C. mediterranea* as a masquerader of the algae, similar to a twig‐resembling caterpillar or leaf‐resembling butterfly (Skelhorn, Rowland, Speed, & Ruxton, 2010). Alternatively, if the sea slug appears cryptic against a large patch of algae, this might be considered as matching its background. The current definitional difference between background matching, masquerade, and mimicry lies in the difference in predators' cognitive perception of the target being a prey (background matching), interfering with recognition of prey as an edible item (masquerade), or mistakenly recognizing the prey as an unpalatable prey item (mimicry). Similar examples in which an animal resembles a larger organism for masquerade, mimicry, or background matching are not limited to visual resemblance. The diet‐induced chemical resemblance to the host plant by the caterpillar (*Biston robustum*) is an example of olfactory crypsis (Akino et al., 2004). The smell of these caterpillars can resemble the cuticular chemicals of their foodplant after a single molt, and this adaptation enables the caterpillars to avoid predation by ants.

To distinguish between background matching and the latter two, attack speeds of naïve and more experienced predators necessitate examination. Since in background matching the prey is simply ignored instead of misidentified, there should be no difference in the attack speed regardless of previous experience. But for a predator that has been previously exposed to the inedible model of the masquerader or mimic, it will take longer and be more cautious when attacking the prey compared to a naïve predator (Mark et al., 2021; Skelhorn, Rowland, Speed, & Ruxton, 2010).

One difference between masquerade and Batesian mimicry is that models of mimicry should try to out‐evolve the mimics, but not in masquerade. Skelhorn, Rowland, and Ruxton (2010, p. 4) wrote, “any change in the population/evolutionary dynamics of the model caused by the presence of the masquerader will not be as a result of the signal receiver changing its behaviour towards the model”. Therefore, using the word “mimic” in both contexts is confusing since it implies mimicry and masquerade have similar evolutionary consequences. Such ambiguity of word use is seen in many studies of stone/tree/leaf masquerade, and researchers often use the phrase such as “leaf‐mimicking” or “twig‐mimicking” insects to mean “leaf‐resembling” masqueraders (Brumfield et al., 1997; Costa et al., 2018; Solano‐Ugalde, 2011; Stoddard, 2012; Wiklund & Tullberg, 2004). Future studies should avoid using the polysemic word “mimic”, but instead, use words that do not represent another defensive mechanism, such as “resemble” or “feature” for masqueraders.

Other authors accept the use of “mimic” when describing masquerade and combine masquerade altogether within protective deceptive mimicry (Ruxton et al., 2018a). The word “mimic” is useful when discussing masquerade as it implies that the animal has evolved to resemble a cue, rather than having a coincidental appearance. Furthermore, when the prey resembles an uninteresting but *living* object (e.g., a living leaf or stick), then masquerade actually overlaps conceptually with Batesian mimicry. This is because the living model, such as a leaf, may receive an increase in attack rate from birds (the signal receiver) due to the presence of the leaf‐resembling comma butterfly (the masquerader) (Wiklund & Tullberg, 2004). Even though this selection pressure may be very small, it is still an increase and could thus be considered Batesian mimicry.

**FIGURE 4:**
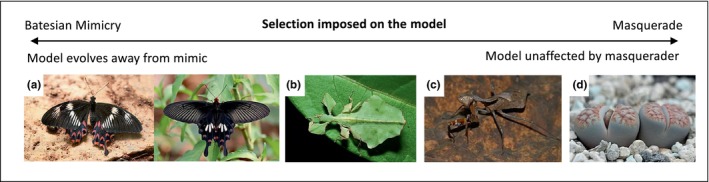
Examples showing the spectrum of selection pressure imposed on the model from Batesian mimicry to masquerade. (a) *Papilio polytes* (left) mimicking unpalatable *Pachliopta arstolochiae* (right), image: Muhammad Mahdi Karim (left) and Atanu Bose Photography (right); (b) leaf insect (*Phyllium gaganteum*) resembling a living leaf, image: Pavel Kirillov; (c) dead leaf mantis (*Deroplatys desiccate*) resembling a dead leaf, image: Bernard Dupont; (d) Karas Mountains living stone (*Lithops karasmontana*) masquerading as a stone, image: Dornenwolf.

This problem has been reviewed by Cuthill (2019), who suggested that there should be a spectrum of selection pressures between Batesian mimicry and masquerade for living models, with Batesian mimics imposing a large selection pressure on the model to evolve away from the mimic, and masquerader imposing no pressure at all (Figure 4). It is no doubt important to distinguish between masquerade and Batesian mimicry, as the prior is “ignored” by the predator and the latter is “avoided” (Cuthill, 2019), but it may be challenging to separate the effects. For example, a bird's feces should be *ignored* as a food item, but should probably also be *avoided* as a food item, so a feces‐resembling caterpillar can be both a masquerader and a Batesian mimic. Debate over the use of these terms is ongoing.

## 4 CONCLUSIONS

Protective defense mechanisms in animals have been extensively documented and can be ordered into different stages of the predatory sequence, each with various mechanisms that prey use to maximize survival. However, there are still a number of examples in nature that do not easily fit into the current conceptualization and need clarification.

The first challenge is the difficulty of identifying protective mechanisms due to conflicting experimental results. We identified just three examples of defensive display which require further experiments (Figure 1): deimatism or Batesian mimicry in the Sphinx moth, lizard tail‐wiggling and deer tail‐flagging behavior which are not well resolved in part because of different study designs. Misunderstanding which defense mechanisms are involved affects the interpretation of predator–prey dynamics and understanding of the evolution of these defense behaviors. In the first example, deimatism could impede avoidance learning in the predator and thus counteract the efficacy of Batesian mimicry. In the second, differential fitness costs associated with pursuit deterrent and deflection could impact both the predator and prey's energy expenditure and the latter's survival probability. A lizard's tail is less likely lost if used as a pursuit deterrent signal compared to being used in deflection, saving energy involved in regrowing a new tail. Third, different underlying selection pressures are involved in perception pursuit deterrence and alarm signaling tail‐flagging in deer.

We also highlighted examples of context‐dependence defense mechanisms where an individual can switch between different mechanisms using the same appearance (Figure 2). We discussed background context, where for example, changes in visual exposure can change the defense mechanism from background matching to masquerade; viewing distance which causes a switch from using camouflage to aposematism; and predator toxin tolerance, which affects whether Batesian, quasi‐Batesian, or Mullerian mimicry might be operating. This emphasizes the importance of considering how multiple parameters may influence the efficacy of different protective mechanisms.

Difficulties can also arise from imprecise language due to polysemy. Terms like flash, mimic, and distract may be incorrectly used to describe behaviors that do not actually serve the intended protective mechanisms, namely flash behavior, mimicry, or distraction. Although using simpler terminology makes articles more accessible to a wider audience, researchers are advised to use precise terminology to prevent confusion.

We also identified three areas where mechanisms have clear definitions but may overlap with other similar mechanisms. (1) Deimatic and flash behavior both involve sudden displays of conspicuous color patches but differ in their effect on the predator. (2) Tonic immobility, freezing, and some forms of aggressive mimicry involve stopping all movements but are performed at different predation stages to achieve distinct purposes. (3) Evasive and locomotor mimicry both involve the movement of the model, but the former involves mimicking the appearance of the hard‐to‐catch model, whereas the latter focuses on mimicking the escape behavior of an unpalatable model. Although significant progress has been made in clarifying these mechanisms to avoid misconceptions with other terms, there are still studies that refer to these pairs of mechanisms interchangeably.

Finally, there are unclear definitions that need addressing. Two sets of mechanisms, pursuit deterrence and masquerade‐Batesian mimicry, require definitions to be reformulated. The definition of pursuit deterrence may need to recognize vigilance, perception and quality advertisement as a gradation of increasing cost (Table 2) and future studies may wish to look at costs. There is an overlap between masquerade and Batesian mimicry in some circumstances with varying consequences for prey–predator coevolutionary dynamics. Likely there are continua for other defense mechanism systems brought about, for instance, by increasing toxicity of the mimic affecting Batesian and Müllerian mimicry and this may affect multiple predators in different ways.

In conclusion, the study of animal antipredator defense mechanisms still demands more precise terminology and clearer definitions given its complexity. To avoid potential confusion, the names of new mechanisms should be carefully crafted to avoid polysemic words that may be applied in other contexts unrelated to defense mechanisms. Clear definitions are also essential to guarantee precision in interpreting experimental findings and definitions should be framed in a manner that is inclusive of all currently known putative examples of defense mechanisms across different taxa.

It is also vital to acknowledge that animals often employ multiple mechanisms that may not be mutually exclusive (Postema et al., 2023); for instance, animals may use a region of color for several functions against different predators (Fabricant & Smith, 2014). Researchers would benefit from designing experiments to test more than one hypothesis when investigating the function of animal defense displays, and so consider how particular defenses can be used in multiple ways and be used differently against several predators.

## AUTHOR CONTRIBUTIONS

**Yuqian Huang:** Conceptualization (equal); writing – original draft (lead); writing – review and editing (equal). **Tim Caro:** Conceptualization (equal); validation (lead); writing – review and editing (equal).

## CONFLICT OF INTEREST STATEMENT

The authors declare no conflicts of interest.

## Data Availability

Data sharing is not applicable to this article as no new data were created or analyzed in this study.
